# From Omics Analysis toward Physiological Mechanism Research in Plants

**DOI:** 10.3390/life13122275

**Published:** 2023-11-29

**Authors:** Yan Lu, Sen Meng, Jie Luo

**Affiliations:** 1 Jiangsu Key Laboratory for the Research and Utilization of Plant Resources, Institute of Botany, Jiangsu Province and Chinese Academy of Sciences (Nanjing Botanical Garden Memorial Sun Yat-Sen), Nanjing 210014, China; 18260093866@163.com; 2 State Key Laboratory of Tree Genetics and Breeding, Research Institute of Tropical Forestry, Chinese Academy of Forestry, Guangzhou 510520, China; 3 College of Horticulture and Forestry Sciences, Hubei Engineering Technology Research Center for Forestry Information, Huazhong Agricultural University, Wuhan 430070, China

With the development of big data in system biology researches, the high-throughput omics analysis has become the most popular high technology in the fields of plant research [1,2]. Increasing studies have applied multi-omics such as genomics, transcriptomics, proteomics and metabolome to uncover the underlying mechanisms of plant physiology, bringing many new discoveries [3,4,5,6]. Undoubtedly, it is significant to combine omics and traditional physiology techniques to explore the insights into plant development, abiotic stresses responses, hormonal and environmental signaling and key trait formation. In this Special Issue, a total of ten papers were collected, including eight research papers and two review papers.

Cutting propagation is an important vegetative propagation method for rapid and large-scale reproduction samplings in agricultural practices [7]. Many plants can form adventitious root easily with cuttings without any treatment, but some plants need special treatments for efficient rooting [8]. Exogenous applications of hormones such as auxins are widely used for stimulating cutting rooting capacities, especially for some difficult-to-root plants [8,9,10]. Quan et al. compared different effects of phytohormones on the rooting capacities of *Catalpa bignonioides* softwood cuttings and found that applications of 1 g L^−1^ IBA significantly promoted rooting rate compared to control and other growth regulator treatments (Contribution 1). The exogenous application of growth regulators also triggered the changes in endogenous hormone levels and ratios in the phloem of *C. bignonioides* softwood cuttings, which might be beneficial for rooting (Contribution 1). In another paper, Sun et al. evaluated the effects of different types and concentrations of hormones as well as the influence of soaking times on the rooting capacities of *Morus* ‘Yueshenda 10′ softwood cuttings and found that applications of 800 mg L^−1^ commercial rooting powder ABT1 for 30 min could significantly improve the rooting ratio up to 66.24% (Contribution 2). These two papers provided a reference to select optimal hormones for stimulating the rooting ratio of softwood cuttings in woody plants.

*Santalum album* L. is a semi-parasitic evergreen tree with important economic value [11]. Taking advantage of the sandalwood transcriptome, Zhang et al. identified eight basic helix–loop–helix (*bHLH*) gene member using bioinformation methods (ontribution 3). The authors further found that SaMYC1 could function as transcription factors and activate the expression of two genes (*SaSSy* and *SaCYP736A167*) that participate in the biosynthesis of the main sandal sesquiterpenes (Contribution 3). Similarly, Chen et al. identified nine plant-specific Rac/Rop small GTPases gene members with the full-length transcriptome data of *S. album* and evaluated the gene expression levels of *Rac* genes in response to drought and hormone treatments (Contribution 4). The authors also identified several key *Rac* genes that closely related to the formation of haustoria in *S. album*, which is important for their semi-parasitic lifestyle (Contribution 4). Due to the lack of complete genome information and effective transgenic methods for *S. album*, a gene functional study could not be applied in *S. album*, which might be a bottleneck to understand the molecular mechanism underlying the growth and environmental adaptations in *S. album* for now. However, with the acknowledgement obtained from the bioinformation analysis combined with molecular techniques, the fundamental molecular mechanisms underlying the synthesis of sandal sesquiterpenes and formation of haustoria will be uncovered soon in the future.

Volatile aroma is an important trait determining the quality of fruits [12]. Taking advantage of RNA-seq, Li et al. conducted a time-series transcriptomic analysis in nine sample stages from fruit development to the fruit storage to uncover the molecular network underlying the volatile aroma formation in pears (Contribution 5). Several key modules related to fatty acid formation were detected using the WGCNA methods, and hub genes in the modules related to the volatile aroma were identified by gene co-expression analysis (Contribution 5).

*Disrupted meiotic cDNA* (*DMC1*) is an important gene that controls DNA recombination through crossing over in meiosis [13]. Kumar et al. applied the RNA interference approach to produce asynaptic mutants by knockdown of the *StDMC1* gene (Contribution 6). The *StDMC1* RNAi lines significantly reduced the pollen viability compared to Kufri Jyoti control plants, with reduced expression levels of the *StDMC1* gene (Contribution 6). *Isodon rubescens* (Hemsl.) Hara is an important medicinal plant in China; its active ingredients show anti-tumor effects [14]. Lian et al. cloned a UDP-glycosyltransferase gene (*UGT*) *IrUGT86A1-like* in *I. rubescens* and predicted the protein properties and cellular location (Contribution 7). The *IrUGT86A1-like* gene was highly expressed in leaves and its expression levels in tissues were highly correlated to the oridonin contents (Contribution 7). Furthermore, gene expression analysis revealed that *IrUGT86A1-like* was positively regulated by NaCl and MeJA treatments, but negatively regulated by abscisic acid (ABA) treatment (Contribution 7). Chen et al. reported that the maize *Constitutively Photomorphogenic 1* (*ZmCOP1*) gene could significantly promote maize mesocotyl lengths and plant height (Contribution 8). By using RNA-seq techniques, the authors identified several different expression genes between B73 and the *zmcop1-1* mutant; pathway analysis indicated the major changes in phytohormone signal transduction (Contribution 8). The results collectively supported that the *ZmCOP1* gene regulates maize architecture by affecting hormone pathways, the underlying mechanisms need to be further explored.

Two review papers were published in this Special Issue. Lin et al. nicely summarized the recent advances of phosphate-binding loop guanosine triphosphatases (P-loop GTPases) in plant growth and stress response (Contribution 9). The authors introduced in detail the structure of the G domain of G proteins, the G domain of YchF, and compared different protein structures (Contribution 9). The critical roles played by YchF in plant oxidative stress, environmental stress response, protein biosynthesis and degradation, and maintaining proteostasis were extensively reviewed by the authors (Contribution 9). miRNAs play crucial roles in plant growth and development, as well as environmental responses. Teng et al. reviewed the recent advances in the transcriptional regulation of miRNAs in plants (Contribution 10). The authors summarized the recent methods used for identifying microRNA promoters, and the important roles played by miRNA in plant growth and development, synthesis of secondary metabolites, disease resistance, abiotic stress, phytohormone signaling pathways, and so on (Contribution 10).

These research papers collectively highlight the significance of applying multiple techniques to understand the mechanisms underlying the physiology of plant development, environmental stresses, and key trait formation. Key genes involved in these physiological processes have been identified, and it is significant for us to build targeted strategies for plant resource protection and utilization. Moreover, these studies encompass a wide array of plant species, mainly including non-timber woody plants, medical plants and crops.

As guest editors, we believe that this Special Issue offers a valuable compilation of research findings that contribute to omics analysis toward physiological mechanism in plants. The diversified species and omics technique outline the potential of combining omics and physiological analyses to enhance plant conservation, sustainable production and utilization. However, more research using innovative omics techniques such as ATAC-seq and single cell RNA-seq to unravel the underlying mechanisms of plant physiology is urgently needed to further expand our knowledge. We thank all contributing authors for their work on this Special Issue.
